# Using path signatures to predict a diagnosis of Alzheimer’s disease

**DOI:** 10.1371/journal.pone.0222212

**Published:** 2019-09-19

**Authors:** P. J. Moore, T. J. Lyons, J. Gallacher, for the Alzheimer’s Disease Neuroimaging Initiative

**Affiliations:** 1 Mathematical Institute, University of Oxford, Oxford, United Kingdom; 2 Department of Psychiatry, University of Oxford, Oxford, United Kingdom; Nathan S Kline Institute, UNITED STATES

## Abstract

The path signature is a means of feature generation that can encode nonlinear interactions in data in addition to the usual linear terms. It provides interpretable features and its output is a fixed length vector irrespective of the number of input points or their sample times. In this paper we use the path signature to provide features for identifying people whose diagnosis subsequently converts to Alzheimer’s disease. In two separate classification tasks we distinguish converters from 1) healthy individuals, and 2) individuals with mild cognitive impairment. The data used are time-ordered measurements of the whole brain, ventricles and hippocampus from the Alzheimer’s Disease Neuroimaging Initiative (ADNI). We find two nonlinear interactions which are predictive in both cases. The first interaction is change of hippocampal volume with time, and the second is a change of hippocampal volume relative to the volume of the whole brain. While hippocampal and brain volume changes are well known in Alzheimer’s disease, we demonstrate the power of the path signature in their identification and analysis without manual feature selection. Sequential data is becoming increasingly available as monitoring technology is applied, and the path signature method is shown to be a useful tool in the processing of this data.

## Introduction

Alzheimer’s disease (AD) is an irreversible brain disorder which progressively affects cognition and behaviour, and results in an impairment in the ability to perform daily activities. It is the most common form of dementia in older people, affecting about 6% of the population aged over 65, and it increases in incidence with age. The initial stage of AD is characterized by memory loss, and this is the usual presenting symptom. Memory loss is one constituent of mild cognitive impairment (MCI), a syndrome in which cognitive decline is greater than is usual for an individual’s age and education level. MCI is diagnosed by complaints of subjective memory loss (preferably corroborated by a close associate or partner of the individual), impairment of memory function, unimpaired general cognition and behaviour, but with no evidence of dementia [1]. A proportion of those diagnosed with MCI will ultimately receive a diagnosis of Alzheimer’s disease, with the yearly rate of progression varying between studies and with the criteria used for defining MCI [2] [3]. In cases where an individual does develop Alzheimer’s disease, the phase of MCI ends with a marked decline in cognitive function as the disease pathology takes effect [1].

The disease leads to an irreversible loss of brain function so there has been much work on predicting a diagnosis of Alzheimer’s disease using a variety of predictor variables. These studies are often motivated by the need to test drug therapies and for the identification of predictive factors which might help in understanding the condition. Input variables for use with machine learning methods may be derived from imaging—in particular structural magnetic resonance imaging (MRI)—cognitive tests, physical biomarkers such as APOE4 status (a known genetic risk factor), and demographic variables such as age and gender [4].

Our purpose is to apply the path signature, a mathematical tool for the selection of machine learning features, to demonstrate its usefulness in a medical context. Time-ordered data is becoming increasingly available as patient monitoring methods are applied using wireless technologies, and it is commonly analysed using machine learning. Features for time-ordered data are typically selected using ad-hoc approaches based on domain knowledge or using by automatic methods based on subset selection or shrinkage [5]. However such approaches can miss interactions between variables or the time ordering of events, features which can be be both predictive and helpful in understanding a disease. Both cases are handled by the path signature, and we show how such interactions might then be interpreted in terms of physiology. Path signatures have successfully been used in feature selection for modelling bipolar disorder and borderline personality disorder [6] as well as other non-medical applications [7] [8]. In this paper we use the path signature method to identify Alzheimer’s disease, and so demonstrate the more general applicability of the method.

## Subjects and methods

Our method is to distinguish two pairs of matched sets using time series sampled over a period of 2 years. The first pair is made up of participants with Alzheimer’s disease *vs*. healthy participants, and the second comprises Alzheimer’s disease *vs*. MCI. In the training data, participants in the AD set all have a first diagnosis of Alzheimer’s disease at 3 years. Participants in the healthy and MCI sets each have their respective diagnoses from the start of monitoring until at least until 6 years afterwards. We use ten-fold cross-validation on the training data to identify the features that are important for prediction, and we evaluate the prediction accuracy on a separate test data set.

### Data sets

A number of competitions have been organized to identify predictors and methods for the automatic diagnosis of Alzheimer’s disease: recent examples are CADDementia [9], the Kaggle Neuroimaging Challenge [10], and the TADPOLE Grand Challenge [4]. Several data repositories are used for competitions and studies, the most comprehensive and widely used being the Alzheimer’s Disease Neuroimaging Initiative (ADNI) [11]. ADNI is comprised of four phases: ADNI-1 (2004), ADNI-GO (2009), ADNI-2 (2011), and ADNI-3 (2016). ADNI-1 registered 200 healthy elderly, 400 participants with MCI, and 200 participants with AD, and subsequent phases continued to add participants. ADNI is led by Principal Investigator Michael W. Weiner, MD; for up-to-date information see www.adni-info.org. Other large scale studies are the Australian Imaging Biomarkers and Lifestyle Study of Ageing (AIBL) which started in 2016 [12], and the European AddNeuroMed collaboration, which was formed for the discovery of novel biomarkers [13]. Overall, ADNI is the most highly cited although there are many other individual studies as well as initiatives which integrate different data sets. Weiner *et al*. [11] lists 49 papers which use the ADNI data in machine learning. Most of these studies used the support vector machine (SVM) as the learning method while three papers used random forests [14–16]. A summary of machine learning for the automatic diagnosis of Alzheimer’s disease is provided in [17]. ADNI data is the basis for the TADPOLE grand challenge https://tadpole.grand-challenge.org/ which was completed in June 2019. For the classification tasks described in this paper, we use the data from the TADPOLE grand challenge.

### Features

Brain imaging methods can be used to derive features for predicting diagnosis either by analysis of the whole brain (voxel-based morphometry) or by deriving features, especially the volume, from brain regions that change during the course of Alzheimer’s disease. Using ADNI data, Schmitter *et al*. [18] found that volume-based morphometry achieved at least as good an accuracy as voxel-based morphometry for classifying Alzheimer’s disease, MCI and controls. Sørensen *et al*. examined the differential diagnosis of AD and MCI using features derived from MRI and found that the most important MRI biomarkers were the hippocampal volume, ventricular volume, hippocampal texture, and parietal lobe thickness. Using training data derived from both the ADNI and AIBL studies, they took first place on the CADDementia challenge with a multi-class classification accuracy of 63% [19]. A variety of features have been identified as predictive of diagnosis: Westman *et al*. used the ADNI and AddNeuroMed data to examine 34 regional cortical thickness measures and 23 volume measures. They found in both cohorts that the most important features were entorhinal cortex, hippocampus and amygdala volumes [20].

Our earlier paper describes the selection of a number of features for predicting Alzheimer’s disease [17]; the method took second place in the competition for the most accurate diagnosis https://tadpole.grand-challenge.org/. In the current study we look beyond the predictive potential of individual regions of interest to that of the relative change in their volumes. The rationale for focusing on the change in relative volumes is that we can potentially detect atrophy in brain regions independently of age-dependent volume reduction. For simplicity we use just three variables from which to derive features: the volumes of the hippocampus, ventricles and whole brain. We include the hippocampus and ventricles volumes because these variables are known to be predictive of diagnosis, and we include the volume of whole brain to find the relative change of these brain regions in comparison.

### Data

**Fig 1:**
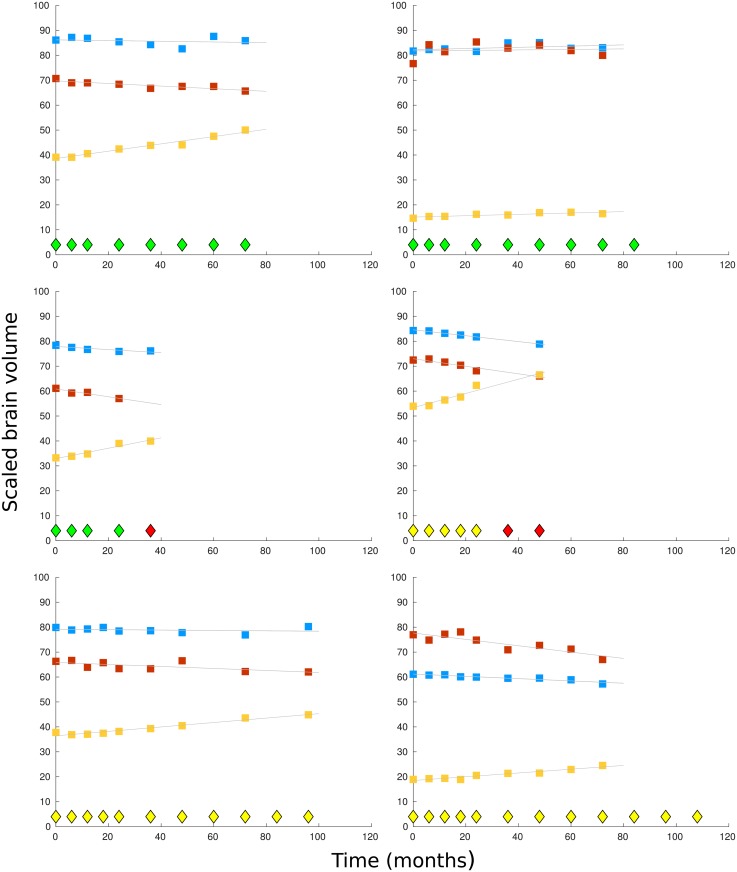
Sample plots by time (months) of scaled brain volumes for 6 participants. Diagnosis points are shown as diamonds at the base of each graph, with a healthy diagnosis shown in green, MCI in yellow, and Alzheimer’s disease in red. In the top two graphs, the participants have a healthy diagnosis throughout monitoring. In the middle two graphs the participants receive a diagnosis of Alzheimer’s disease 3 years after monitoring began. In the bottom the two graphs, the participants have a diagnosis of mild cognitive impairment throughout monitoring. The scaled brain volumes are shown in each graph from the top down as: whole brain (blue markers), hippocampus (red markers), and ventricles (yellow markers).

A total of 1737 participants from the TADPOLE data set are first split into those who have a diagnosis of Alzheimer’s disease at some time (AD, n = 688), those who always have a healthy diagnosis (NL, n = 424), and those who always have an MCI diagnosis (MCI, n = 484). Both the training and test data is selected from these three sets, so we compare participants whose diagnosis converts to Alzheimer’s disease with those whose diagnosis remains unchanged. The training data is found by selecting participants with a first diagnosis of Alzheimer’s disease at 36 months from baseline (start of monitoring) and with at least four measurements of all the variables WholeBrain, Hippocampus and Ventricles in the 24 months since baseline, with a measurement at 24 months. Measurements in this period are available at 0, 3, 6, 12, 18 and 24 months; there are only a few measurements at month 3, which we exclude from the analysis, and there is some missing data at 18 months. Each participant with Alzheimer’s disease must have matching counterparts in both the NL and MCI sets. For an individual with an NL/MCI diagnosis to qualify as a counterpart they must match the age of the AD individual to within 5 years and their diagnosis must remain unchanged for the 72 months since their first (baseline) measurement. The matching time series must also have at least four measurement points up to month 24, again including a measurement at month 24. Sample plots of the time series are shown in Fig 1 and characteristics of the sets selected for training are shown in Table 1.

**Table 1:** Demographic, physical and genetic characteristics for the training data. The variables shown are the sample size *n*, age, gender, scaled medians(iqr) of MRI volume measurements at baseline, and APOE4 status. In the AD set, 19 participants transition from MCI, while 2 participants transition from a healthy diagnosis.

	*Healthy (NL)*	*Alzheimer’s disease(AD)*	*MCI (MCI)*
*n:*	21	21	21
*Age: min*	65.1	64.6	60.7
*mean*	75.2	75.4	74.7
*max*	89.6	85.9	83.8
*Gender M/F:*	8/13	10/11	13/8
*Wholebrain:*	1.02 (0.13)	1.00 (0.12)	1.00 (0.12)
*Hippocampus:*	7.21 (1.23)	6.14 (1.41)	7.10 (0.89)
*Ventricles:*	28.53 (18.55)	34.34 (21.17)	33.88 (26.78)
*APOE4 0:*	81%	29%	71%
*1:*	19%	52%	29%
*2:*	0%	19%	0%

The participants in the test data are separate from those in the training data, but like the training data they are formed into three subsets (AD, MCI and NL). The test data makes use of time points starting from 12 months since the first baseline measurement. It comprises participants who have a first diagnosis of Alzheimer’s disease at 48 months (AD, n = 10), and participants with a healthy diagnosis (NL, n = 20) and a diagnosis of mild cognitive impairment (MCI, n = 6) where the NL or MCI diagnosis is maintained for at least 84 months since baseline. The NL and MCI subsets in the test data are not matched to the AD set since they would not be matched in a real world use of the classifier. The measurements used for analysis are at 12, 24 and 36 months for all three sets (AD, NL, MCI), sample times which are different from that for the training data, but the path signature generates the same length of feature vector in either case.

### Path signature

The path signature is a systematic way of providing feature sets for sequential data that can encode nonlinear interactions in the data as well as giving linear features [7, 8]. Combined with Lasso regularisation [21] it provides a set of features that can significantly improve the inference when nonlinear interactions are important: since the signature encodes all time-dependent interactions it can detect unforeseen relationships between variables. Second order information has proved useful in some applications, for example in [6] where the method was used to model bipolar disorder and borderline personality disorder.

The path signature was originally introduced by Chen [22] who applied it to piecewise smooth paths, and it was further developed by Lyons and others [7, 23–26]. It is defined as follows: a path *X* through a space $R^{d}$ is a continuous mapping from an interval [*a*, *b*] to $R^{d}$. The path is dependent on parameter *t* ∈ [*a*, *b*], and can be written,

$$\begin{array}{c} X_{t}=\left\{X_{t}^{1},X_{t}^{2},X_{t}^{3},\ldots X_{t}^{d}\right\} \end{array}$$ (1)

The kth-fold iterated integral of X is given by,

$$\begin{array}{c} S{\left(X\right)}_{a,t}^{i_{1},\ldots i_{k}}=\int_{a<t_{k}<t}\ldots \int_{a<t_{1}<t_{2}}dX_{t_{1}}^{i_{1}}\ldots dX_{t_{k}}^{i_{k}} \end{array}$$ (2)

The path signature is a collection of all the iterated integrals of *X*,

$$\begin{array}{c} S{\left(X\right)}_{a,b}=\left(1,S{\left(X\right)}_{a,b}^{1},S{\left(X\right)}_{a,b}^{2},S{\left(X\right)}_{a,b}^{1,1},S{\left(X\right)}_{a,b}^{1,2},\ldots \right) \end{array}$$ (3)

*S*(*X*)_*a*,*b*_ is a series of real numbers, and the superscripts *a*, *b* are drawn from the set *G* of all multi-indexes,

$$\begin{array}{c} G=\{\left(i_{1},\ldots ,i_{k}\right)|k⩾1,i_{1},\ldots ,i_{k}\in \left\{1,\ldots ,d\right\}\} \end{array}$$ (4)

In effect the path signature transforms multivariate sequential data (which may have missing or irregularly sampled values) into a finite length series of real numbers which uniquely represents a trajectory through Euclidean space. The length of the path signature depends on the number of input variables and the degree, which is the order *k* of the highest order iterated integral. So in two dimensions a path signature of degree two is *S* = {1, *S*^(1)^, *S*^(2)^, *S*^(1,1)^, *S*^(1,2)^, *S*^(2,1)^, *S*^(2,2)^} while a path signature of degree 3 would include the terms *S*^(1,1,1)^, *S*^(1,1,2)^ etc.. A more detailed introduction to the path signature which also gives a geometric interpretation of the terms can be found in [8].

### Example

**Fig 2:**
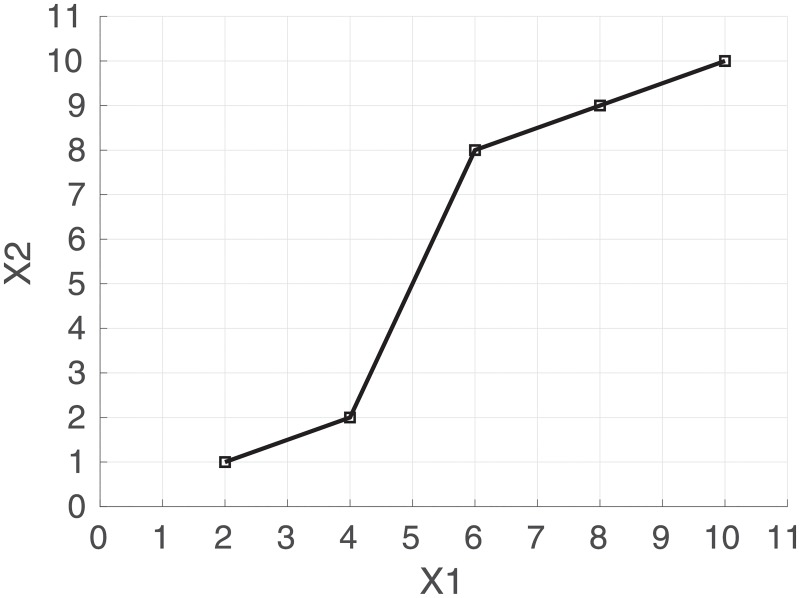
Path of a trajectory through $R^{2}$ where *X*1 = {2, 4, 6, 8, 10} and *X*2 = {1, 2, 8, 9, 10}. The path signature of degree 2 is {1, 8, 9, 32, 31, 41, 40.5}.

An example path is shown in Fig 2 where the space is $R^{2}$ and the variables are named *X*1 and *X*2.

**Fig 3:**
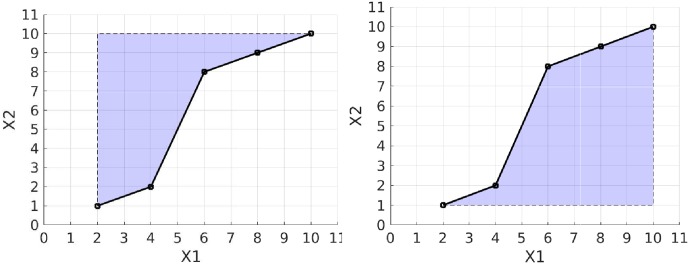
Geometric interpretation of the cross terms in the signature using the same path as in Fig 2. The graph on the left shows the shaded area representing *S*^(1,2)^ with value 31 and the graph on the right shows *S*^(2,1)^ where the shaded area has a value of 41. These terms both measure the deviation from a straight line and they distinguish cases where X1 increases quickly relative to X2 and vice versa.

The first term of the path signature is always 1 by convention, and the next two terms are *S*^(1)^ = 8 and *S*^(2)^ = 9 which are the increments in dimensions *X*1 and *X*2 respectively from the start to the end of the path. The next term is *S*^(1,1)^ = 32, which is equal to $\frac{1}{2}{\left(S^{\left(1\right)}\right)}^{2}$. The cross terms, *S*^(1,2)^ = 31 and *S*^(2,1)^ = 41, each represent areas between the actual path and a stepped path as shown in Fig 3. The final term is *S*^(2,2)^ = 40.5 which is equal to $\frac{1}{2}{\left(S^{\left(2\right)}\right)}^{2}$.

**Fig 4:**
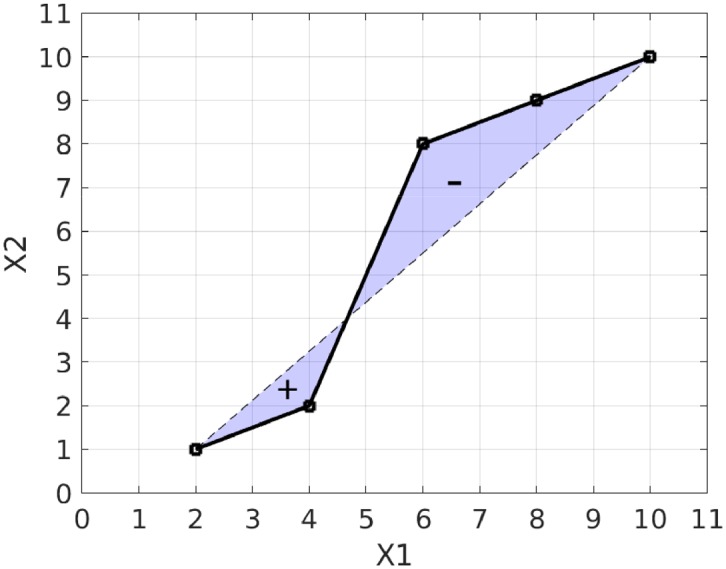
Geometric interpretation of an area term in the log signature. The term has an area equal to the upper shaded region subtracted from the lower shaded region which in this example evaluates to -5. The log signature has a single term for the area between the path and its chord whereas the signature uses two area terms. The log signature of degree 2 in this example is {8, 9, −5}.

It is apparent from the example that there is some redundancy in the path signature terms: for example, *S*^(1,1)^ is equal to $\frac{1}{2}{\left(S^{\left(1\right)}\right)}^{2}$, and the shaded areas in Fig 3 are complementary. By taking the logarithm in the formal power series shown above we obtain a more compact representation, the log signature. In the example of Fig 2 the log signature is {8, 9, −5}. In this case there is no leading 1, and the two terms with values 8 and 9 each represent the increment of each variable as they do in the path signature. The next term has value −5 and it represents an area term measuring the deviation from a straight line as shown in Fig 4. It is found from the two areas between path and chord, and it is equal to the area below the chord minus the area above the chord.

### Signature terms

The experiment in this paper uses four variables (1:WholeBrain, 2:Hippocampus, 3:Ventricles, 4:Time) and a path signature of degree two. The path signature and log path signature are formed of the following sequences, where bracketed terms represent variables and their combinations.

- Path signature: [1, (1), (2), (3), (4), (1,1), (1,2), (1,3), (1,4), (2,1), (2,2), (2,3), (2,4), (3,1), (3,2), (3,3), (3,4), (4,1), (4,2), (4,3), (4,4)]
- Log path signature: [(1), (2), (3), (4), [1,2], [1,3], [1,4], [2,3], [2,4], [3,4]]

For example the bracketed term (1) represents the increment of the Whole Brain volume, and (2,3) is the area term for the Hippocampus *vs*. Ventricles path. For the log signature, the term [2,3] is the area term for the Hippocampus *vs*. Ventricles path.

### Machine learning

**Fig 5:**
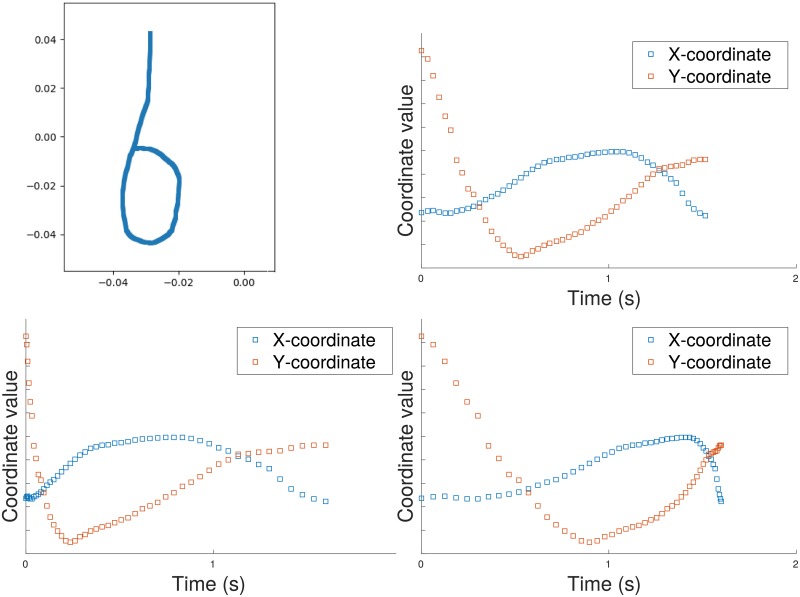
Invariance of the path signature under time reparameterisation. Top left: a path generated by drawing the letter ‘b’ from the top down. Top right: original time series generated by regular sampling of the X- and Y- coordinates of the path. Bottom left: speed increased at start of drawing. Bottom right: speed increased at the end of drawing. In contrast to features derived from the time series, the signature uniquely characterizes the original path. The path signature of degree 2 is {1, −0.0013, −0.0464, 0.0000, 0.0007, −0.0006, 0.0011}.

In machine learning tasks which use sequential data it is common to derive features from time series [5] [27]. Time series representations are dependent on the sampling function, and prediction methods usually assume regular sampling. The path signature representation makes no such assumption; here we demonstrate its invariance to parameterization (in this case the parameter is time), and its robustness to missing values. Fig 5 illustrates a simple path (the letter ‘**b**’ on the top left) followed by some time series representations derived from it by sampling X- and Y- coordinates at intervals. On the top right of the figure is shown the original time series, generated by regular sampling of the pen position. The graphs on the bottom left and bottom right of Fig 5 show a reparameterisation of the original time series obtained by simulating a change in drawing speed. While these time series graphs depend on the speed of the pen, the path signature is computed directly from the original path using both variables X and Y. This parameter invariance property was exploited in a recent project on recognising Chinese handwriting which gave the most accurate results on two standard benchmarks [7].

In Fig 5 the speed of drawing is not important for recognising the shape, but in applications where time is an independent variable, such as the diagnosis of Alzheimer’s disease, it can be added to the space as another dimension in which the path can move. The robustness of path signatures to irregular sampling can be seen from Fig 4 where the area does not depend on the sampling regularity except in that additional samples may provide greater precision for the path representation.

Implementing path signatures for machine learning is straightforward: a Python package called *esig* is provided in the Python Package index https://pypi.org/project/esig/, and its associated documentation explains how to derive the signature from input variables.

### Classification

The training data is used to fit the model whose features we examine, and the test data is used for error estimation. We classify the training data in two tasks, Alzheimer’s disease against a healthy diagnosis (AD *vs*. NL), and Alzheimer’s disease against MCI (AD *vs*. MCI). For classification we use binary logistic regression which models the log probabilities of the outputs as linear functions of the inputs. We use logistic regression for classification because its function can be easier to understand than more sophisticated machine learning methods such as the random forest or neural network. Since the path signature encodes nonlinearity into the features, a simple classifier can immediately reveal the importance of nonlinear effects. The input features are selected using Lasso regularisation, a shrinkage method which subtracts an *L*_1_ penalty from the negative log-likelihood when fitting the model. The complete feature vector prior to selection is formed from the three variables, WholeBrain, Hippocampus and Ventricles, concatenated with the path signature which is itself derived from these variables with their time points. In the classification tasks below we show results for both for the path signature and the log path signature. In both cases the first 7 entries in the feature vector are the same: the first 3 being the baseline values of the variables, entry 4 is the time increment (approximately 24 months for all participants), and entries 5-7 are the increment of each of the variables.

Classification is performed between the sets each containing 21 time series, with diagnosis as the output variable. Training uses 10-fold cross-validation to find the graph of deviance against the Lasso coefficient λ which determines the strength of regularisation. As the Lasso coefficient increases, most of the variable coefficients shrink to zero, leaving a set of variables which act as predictors.

## Results and discussion

### Training data

**Fig 6:**
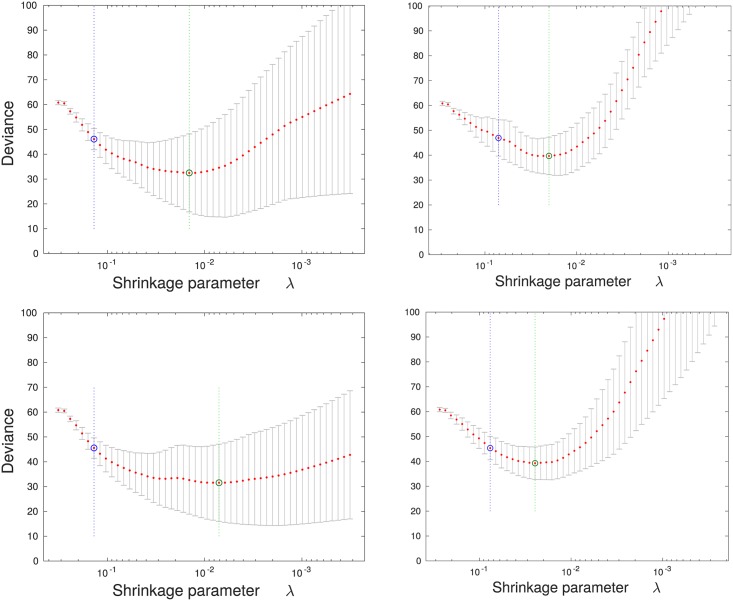
Training curves for classification of Alzheimer’s disease *vs*. a healthy diagnosis (left column) and Alzheimer’s disease *vs*. MCI (right column). The graphs on the top row arise from when the path signature method is used to derive features, and the graphs on the bottom row from the log signature method. In each case the graph shows the optimisation of the Lasso shrinkage parameter λ using 10-fold cross validation, where the value of λ increases from right to left. Deviance is a measure of the estimate of the expected negative likelihood of the parameter applied to new data. The green circle and vertical line locate the minimum cross validation error, and the blue circle and vertical line to the left locate the value of λ with minimum cross-validation error plus one standard deviation.

The training curves for the two classification tasks, AD *vs*. NL and AD *vs*. MCI, are shown in Fig 6. As the value of the shrinkage parameter λ is increased (from right to left in each graph), the variable coefficients shrink and the deviance decreases to a minimum and then begins to increase. On each graph the value of λ at the minimum is marked by a green line to the right, and the value within one standard deviation of the minimum deviance is shown as a blue line on the left.

Table 2 shows the features that are selected at the point of minimum deviance plus one standard deviation. When the path signature is used the following terms are selected for both classification tasks (AD *vs*. NL and AD *vs*. MCI): the baseline hippocampus volume, the rate of change in hippocampus volume, and the change in hippocampus volume relative to the whole brain. Selection of the baseline hippocampus volume is unsurprising: inspection of Table 1 shows that the hippocampus is on average smaller for the AD set compared with the NL and MCI sets. The selection of hippocampus shrinkage rate is consistent with the finding that hippocampal atrophy is predictive of a diagnosis of Alzheimer’s disease [28]. More interestingly, the second order term (Hippocampus, Wholebrain) is found to be predictive. This result most likely reflects accelerated atrophy of the hippocampus relative to the whole brain volume for people with Alzheimer’s disease.

**Table 2:** The set of variables selected by Lasso for classification of Alzheimer’s disease *vs*. a healthy diagnosis (left column) and Alzheimer’s disease *vs*. MCI (right column). Highlighted rows show variables that are selected by both classification tasks. The baseline value of a variable is denoted by the suffix _BL. First order terms such as (Incr. Ventricles) denote the increment of a variable and second order terms such as (Hippocampus, Time) are area terms. Highlighted rows show features selected in both classification tasks.

*Alzheimer’s disease* vs. *Healthy*	*Alzheimer’s disease* vs. *MCI*
*Signature*	
Hippocampus_BL	Hippocampus_BL
	Ventricles_BL
(Incr. Ventricles)	
(Hippocampus, Time)	(Hippocampus, Time)
(Hippocampus, Wholebrain)	(Hippocampus, Wholebrain)
	(Time, Ventricles)
	(Hippocampus, Hippocampus)
*Log signature*	
Hippocampus_BL	Hippocampus_BL
	Ventricles_BL
[Incr. Hippocampus]	[Incr. Hippocampus]
[Incr. Ventricles]	[Incr. Ventricles]
	[Time, Ventricles]

**Fig 7:**
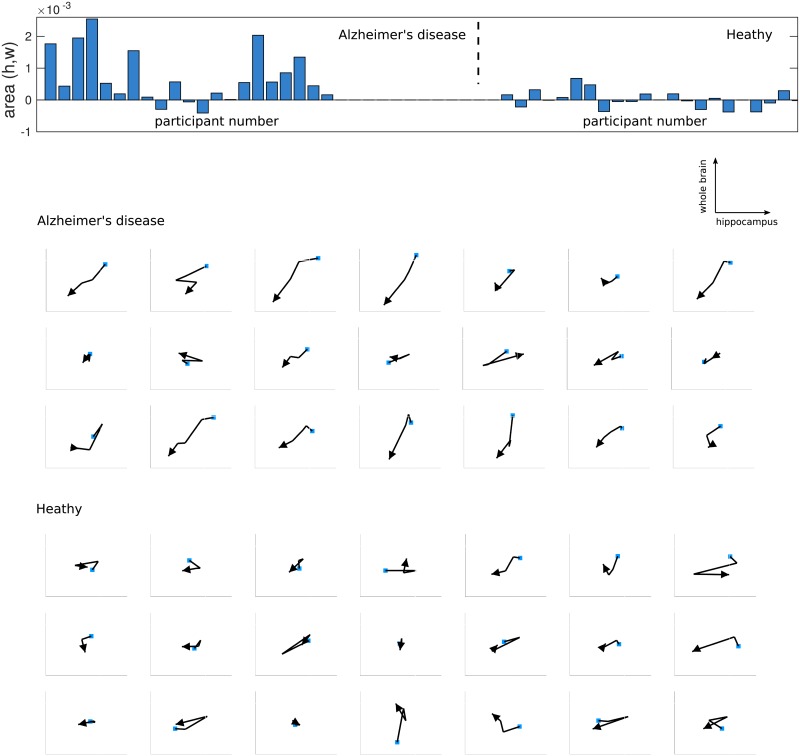
Area terms for the two sets of participants, Alzheimer’s disease and those who remain healthy, with their corresponding paths. The bar chart at the top shows the value of the area (Hippocampus, Wholebrain) path signature term for each set. The graphs show the corresponding paths for each participant in the same order as in the bar chart. The paths are in the scaled hippocampus *vs*. wholebrain space and ordered in time, with the first observation shown by a blue square and the final observation shown by an arrow. Paths directed towards the north-east or south-west generate positive areas; those towards the north-west or south-east correspond to negative areas. The set of participants with Alzheimer’s disease shows a consistent south-west direction in the trajectory; the healthy set has participants with both positive and negative area terms and shows no general trend in the path trajectory.

Fig 7 shows the values of the (Hippocampus, Wholebrain) signature terms for both the Alzheimer’s disease and healthy sets as a bar chart. Time series from the Alzheimer’s disease set have generally large positive values while those from the healthy set have smaller values which are both positive and negative in sign. The figure also shows paths of the trajectory though the hippocampus *vs*. wholebrain volume space, where a longer path in a consistent direction gives a larger area value. Most participants in the Alzheimer’s disease set exhibit a decrease in both hippocampus and whole brain volumes with time, in contrast to the healthy set. While this trend might in principle be found from a combination of increment terms for the two variables, the area term encodes the information into a single feature. Whereas the increment terms use only the first and last measurements, the area term uses all the samples in the path, and it can distinguish different path trajectories such as when one variable changes before a second variable.

For the log signature, the baseline volume of the hippocampus and the increments in hippocampus volume and ventricles volume are selected. The log signature finds fewer second order terms and instead uses the incremental change in the hippocampus and ventricles size as predictors. We note however that using a linear classifier such as logistic regression with the log signature is not optimal, because it requires a non-linear function to ‘unwrap’ the encoded non-linearities. The training data is small, so some of the features might be due to minor differences between the sets resulting from the selection process rather than from differences in the sampled populations.

### Test data

For both classification tasks, Alzheimer’s disease *vs*. a healthy diagnosis, and Alzheimer’s disease *vs*. MCI, we train the model on the training data and estimate the error on the test data in each case. The threshold for choosing the output class is estimated from the ROC curve, and the output from the model is compared with this threshold to generate the binary output. The confusion matrix for the test data prediction is shown in Table 3.

**Table 3:** Confusion matrices for classifying diagnoses in the test data. Left: Alzheimer’s disease *vs*. a healthy diagnosis. Right: Alzheimer’s disease *vs*. MCI. The labels are as follows, NL: healthy, MCI: mild cognitive impairment, AD: Alzheimer’s disease.

*Predicted*						*Predicted*					
		AD	NL	Total	Accuracy			AD	MCI	Total	Accuracy
*Actual*	AD	9	1	10	0.90	*Actual*	AD	9	1	10	0.90
	NL	1	19	20	0.95		MCI	0	6	6	1.00

For the first task, Alzheimer’s disease *vs*. a healthy diagnosis, there are 10 participants in the Alzheimer’s disease set and 20 in the healthy set, and of these just two are misclassified, one in each set. For the second task Alzheimer’s disease *vs*. MCI there are there are 10 participants in the Alzheimer’s disease set and 6 in the MCI set. In this case just one participant in the Alzheimer’s disease set is misclassified, and all the participants in the MCI set are correctly identified. The same result is found using either the signature or the log signature feature set. Without a larger sample, the test accuracy cannot safely be generalized to other data. However, the high accuracy on unseen test data is evidence that the model has not overfitted to the training data.

### Discussion

The path signature has been used to encode interactions between variables to predict those who will receive a diagnosis of Alzheimer’s disease after a period of 3 years. We found two combinations of variable which distinguish both Alzheimer’s disease from healthy individuals, and Alzheimer’s disease from individuals with MCI. These interactions are between the hippocampus volume and time, and between the hippocampus volume and whole brain volume. A change in hippocampus volume with time is known to be predictive of a diagnosis of Alzheimer’s disease. The importance of a relative change between the hippocampus volume and the whole brain volume is interesting. Since some brain atrophy occurs in normal aging it is useful to examine the relative change of brain regions, and the path signature approach is a principled approach to this analysis. We examined the graphs of hippocampus volume *vs*. whole brain volume to illustrate the way this ratio changes over time and show the effectiveness of the signature in providing a feature for prediction. Healthy participants show no systematic decline in the two volumes, while those with Alzheimer’s disease often show a systematic downward trend. The area terms clearly summarise this trend along with any nonlinearity in the path.

A major limitation of the analysis is the small selected sample size (n = 42) for both of the classification experiments, so some of the selected features might be artifacts of the selection procedure. The relatively small amount of data and a short period of monitoring is due in part to the nature of the condition: Alzheimer’s disease progresses over decades, and to our knowledge only the ADNI project has monitored large numbers of individuals over this period of time. Another application of the method using the more recent ADNI-3 data would provide a clearer assessment of the potential value of the method for this application.

The signature method generates interpretable nonlinear features and handles missing and irregular sequential data. Manual feature selection can be used to identify known predictors such as hippocampal and other brain volume changes. However, the path signature provides a systematic way of generating features from time series which can then be selected using shrinkage or other automatic methods. While nonlinear classifiers such as neural nets and random forests often provide accurate predictions, their function can be more difficult to understand than simpler methods. By encoding nonlinearity into the features, we can use simpler classifiers that give more interpretable results and relate feature importance to physiology. Overall, sequential data is becoming increasingly available as monitoring technology is applied, and the path signature method is a useful tool in the processing of this data.
